# Analysis of the surgical approach in prostate cancer staging: results from the surveillance, epidemiology and end results program

**DOI:** 10.1038/s41598-023-37204-y

**Published:** 2023-06-19

**Authors:** Felipe Andrés Cordero da Luz, Camila Piqui Nascimento, Eduarda da Costa Marinho, Pollyana Júnia Felicidade, Rafael Mathias Antonioli, Rogério Agenor de Araújo, Marcelo José Barbosa Silva

**Affiliations:** 1 Center for Cancer Prevention and Research, Uberlandia Cancer Hospital, Av Amazonas nº 1996, Umuarama, Uberlândia, Minas Gerais CEP: 38.405‑302 Brazil; 2 Laboratory of Tumor Biomarkers and Osteoimmunology, Department of Immunology, Institute of Biomedical Sciences, Federal University of Uberlandia, Av Pará nº 1720, Bloco 6T, Room 07, Umuarama, Uberlândia, Minas Gerais CEP: 38.405‑320 Brazil; 3 Medical Faculty, Federal University of Uberlandia, Av Pará nº 1720, Bloco 2U, Umuarama, Uberlândia, Minas Gerais CEP: 38.400‑902 Brazil

**Keywords:** Prostate cancer, Surgical oncology

## Abstract

Surgery is not used as a criterion for staging prostate cancer, although there is evidence that the number of analyzed and affected lymph nodes have prognosis value. The aim of this study was to determine whether there are significant differences in staging criteria in patients who underwent prostatectomy compared to those who did not, and whether the number of affected and analyzed lymph nodes (LN) plays a prognostic role. In this retrospective study, a test cohort consisting of 404,210 newly diagnosed men with prostate cancer, between 2004 and 2010, was obtained from the 17 registries (Nov 2021 submission); a validation consisting of 147,719 newly diagnosed men with prostate cancer between 2004 and 2019 was obtained from the 8 registries (Nov 2021 submission). Prostate cancer-specific survival was analyzed by Kaplan–Meier curves, survival tables and Cox regression; overall survival was analyzed only to compare Harrell's C-index between different staging criteria. In initial analyses, it was observed that the prognostic value of lymph node metastasis changes according to the type of staging (clinical or pathological), which is linked to the surgical approach (prostatectomy). Compared with T4/N0/M0 patients, which are also classified as stage IVA, N1/M0 patients had a shorter [adjusted HR: 1.767 (1429–2184), *p* < 0.0005] and a longer [adjusted HR: 0.832 (0.740–0.935), *p* = 0.002] specific survival when submitted to prostatectomy or not, respectively. Analyzing separately the patients who were submitted to prostatectomy and those who were not, it was possible to obtain new LN metastasis classifications (N1: 1 + LN; N2: 2 + LNs; N3: > 2 + LNs). This new (pathological) classification of N allowed the reclassification of patients based on T and Gleason grade groups, mainly those with T3 and T4 disease. In the validation group, this new staging criterion was proven to be superior [specific survival C-index: 0.908 (0.906–0.911); overall survival C-index: 0.788 (0.786–0.791)] compared to that currently used by the AJCC [8th edition; specific survival C-index: 0.892 (0.889–0.895); overall survival C-index: 0.744 (0.741–0.747)]. In addition, an adequate number of dissected lymph nodes results in a 39% reduction in death risk [adjusted HR: 0.610 (0.498–0.747), *p* < 0.0005]. As main conclusion, the surgery has a major impact on prostate cancer staging, mainly modifying the effect of N on survival, and enabling the stratification of pathological N according to the number of affected LN. Such a factor, when considered as staging criteria, improves the prognosis classification.

## Introduction

The staging of prostate cancer has been progressively refined due to the incorporation of new factors and the reclassification of some others already incorporated, such as PSA, Gleason grade group, tumor size (T) and the presence of regional lymph node (N) or distant (M) metastasis^1,2^.

In the eighth edition of the American Joint Committee on Cancer (AJCC) staging manual, stage IV was subdivided according to the type of metastasis (regional lymph node—N, or distant—M), with a downstaging of T4(N0/M0) disease to either IIIB or IIIC, according to Gleason grade group^1,2^.

However, it is known that the dissection of an extensive amount of pelvic lymph nodes results in the better staging of lymph node metastasis, despite the tendency to reduce the number of lymph nodes dissected due to the issue of improving comorbidities and quality of life^3^. In this regard, there is evidence that the characteristics of the disease, which include the number of affected lymph nodes, imply a different response to adjuvant treatments such as radiotherapy, with evidence favoring total irradiation of the pelvis associated to hormone therapy in patients with more aggressive diseases, but with response limited mainly by the number of affected lymph nodes^4–9^.

The present study aims to investigate whether surgery has a relevant impact on prostate cancer staging.

## Material and methods

Retrospective observational study based on the Surveillance, Epidemiology and End Results (SEER) program [17 registries, Nov 2021 (2000–2019)] database, enrolling prostate cancer patients treated between 2004 and 2015. Validation was performed with data of patients obtained from the SEER program from 8 registries [Nov 2021 (1975–2019)] database. The database was analyzed with ID 14659-Nov2021. Cancer-specific survival was measured as the main outcome (endpoint) and overall survival as secondary endpoint. The latter was used only to compare Harrell's C index between staging criteria.

### Selection criteria

Patients who were 40 years of age or older and had only one primary prostate cancer diagnosis were included.

For the first cohort, the following exclusion criteria were used: a PSA level greater than or equal to 98 ng/mL; no information regarding the reason for the absence of surgery; autopsy- or death certificate-only diagnosis; diagnosis without histopathological confirmation; histology (ICD-O-3) with less than 100 cases; death prior to the recommendation of surgery; the refusal of surgery; unknown whether surgery was performed; unknown whether surgery was recommended; neoadjuvant treatment; follow-up period of less than 1 day; missing staging; stage 0 or occult cancer; absence of sociodemographic information on race, median household income, type of housing region (rural–urban).

Exclusion criteria for the second cohort are as follows: invalid PSA level; invalid information about lymph node analysis or metastasis; no information regarding the reason for the absence of surgery; follow-up period of less than 1 day; stage 0 or occult cancer; insufficient clinicopathological data for staging in both AJCC 8th edition and new staging proposal.

The rationale for the selection of patients for each objective is depicted in Supplementary Fig. 1.

### Classifications

Definitive surgery and pathological Gleason grade group were considered only in the context of prostatectomy and classified as described in the eighth edition of the AJCC. In the absence of the surgical sample, the biopsy was analyzed. Patients diagnosed between 2010 and 2015 with sufficient data were classified according to the eighth edition of the AJCC^1^.

### Statistical analysis

Survival analyses (Kaplan–Meier curve, survival tables, Log-Rank survival distribution test, Cox regression, and time-dependent Cox regression), regression models (Negative binomial and binary logistic), association tests (Pearson’s χ^2^ and Cramer’s V) and correlations (Spearman’s rho and Kendall’s Tau-b) were performed in IBM SPSS v25.0, JAMOVI v. 2.2.5, and MedCalc v. 20.116. The significance level (α) considered was 0.05. Survival models and tests were adjusted for specific survival as the primary outcome.

Prior to the analysis of independent prognostic factors by Cox regression, these were evaluated for proportionality of risks by the Kaplan–Meier (KM) estimator curve associated with the Log-Rank test. For continuous variables, the proportionality of risks was similarly analyzed after categorizing them into predetermined groups and by correlating the partial residuals, generated after univariate regression, with the observation time. In violating risk proportionality by continuous variables, their categorized forms were used. In the breakdown of risk proportionality by categorical variables, time was categorized as the moment of inflection, and the time-dependent covariate (T_COV) with interaction (*) of the moment of inflection was used. Univariable analysis of prognosis factors in a time-dependent context was carried out in the presence of the T_COV* variable.

The Cox regression model with the maximum prognosis (optimization of model) value was obtained using Forward Stepwise Likelihood Ration model with an entry *p*-value equal to 0.10 and output *p*-value equal to 0.05 to reduce covariate collapsibility and overfitted models.

The association between interdependent categorical factors was evaluated using Pearson’s χ2 test. The association was considered positive (direct) when the adjusted standardized residuals had a value > (+ 2.0) and considered negative (indirect/inverse) when the value was < (− 2.0). The interpretation of the effect size of the associations was analyzed with Cramer’s V coefficient, adjusted by the degrees of freedom.

The binary and multinomial logistic regression models were elaborated using the Forward stepwise method (Likelihood Ratio) with an entry *p*-value equal to 0.10 and output *p*-value equal to 0.05. The proportionality/linearity with logits was analyzed by comparing the differences in the coefficients (βs) of the categories of the categorical variables and by the Box-Tidwell transformation for continuous variables. Continuous variables that break the assumption of significance in the insertion of the transformed variable [ln(variable)*variable] were categorized. Categories (levels) with overlapping confidence intervals or that break proportionality were collapsed with similar ones.

In order to analyze factors associated with a higher incidence rate of positive lymph nodes, a generalized linear model for counting variables with a log link was developed. The choice of distribution type was based on the analysis of the dispersion between observed and expected (deviance − value/df), Pearson's χ^2^ and the AIC. The Poisson model was chosen because of its better model deviance.

### Ethics approval

To access and use SEER data, neither the institutional review board review nor the patient informed consent is required.

## Results

### Lymph node metastasis and prostatectomy: restaging analysis depending on surgery and lymph node involvement

A total of 404,210 patients were included in the first cohort, with a median follow-up of 101 months (0–191) and 99,159 deaths recorded, of which 33.9% (n = 33,622) were due to prostate cancer.

Analyzing stage IV criteria according to the seventh edition of AJCC, an antagonistic prognosis value of the T4 and N1 as a function of surgery could be observed (Supplementary Fig. 2A–D). Analysis of time-dependent Cox regressions revealed that the N1/M0 factor was related with decreased survival in prostectomized patients compared to T4/N0/M0 [adjusted HR: 1.767 (1.429–2.184), *p* < 0.0005]. The opposite was observed in those who did not have the prostate removed [adjusted HR: 0.832 (0.740–0.935), *p* = 0.002].

In prostectomized patients with N + disease, the number of affected lymph nodes was an independent prognosis factor [HR: 1.14 (1.11–1.17), *p* < 0.001], with optimal cutoff of > 2. However, prognosis segregation in those with more than two metastatic lymph nodes was observed [HR: 1.08 (1.04–1.013), *p* < 0.001, cutoff > 5], but Cox regression analyses (Supplementary Table 1) and survival analyses stratified by tumor size (not shown) showed that the division into N1(1+), N2(2+) and N3(> 2+) results in better prognosis segregation (Supplementary Fig. 3). Although the prognosis value of N depends on tumor size (Supplementary Tables 2 and 3), patients with T2 or T3 and N1 diseases have a similar cumulative survival rate (92.7% and 86.8%, respectively) to their N2 counterparts (92.0% and 84.4%, respectively). Patients with T4 and N2 disease have cumulative survival rates like their N3 counterparts (58.5% and 62.9%, respectively).

Regarding non-prostectomized patients, we tested which factors can improve the current staging system. Patients staged as IIIB to IVB with T4 tumors had significantly lower specific survival (Supplementary Fig. 4A). This impact allowed patients with IIIB/T3 disease to be downstaged to IIC. However, those with IIIC/T4 disease must be upstaged, making it necessary to segregate patients with IIIC/T1–T3 disease in an exclusive category (Supplementary Fig. 4B).

By time-dependent Cox regression, non-prostectomized patients with IIIB/T4 disease had shorter survival than those with IIIB/T3 [adjusted HR: 4.090 (2.823–5.925), *p* < 0.0005] and IIIC/T1-T3 [adjusted HR: 1.686 (1.234–2.304), *p* = 0.001] diseases. Patients with IVA/T1-T3 diseases [adjusted HR: 1.948 (1.557–2.438), *p* < 0.0005] survived longer than patients with IVA/T4 disease [adjusted HR: 0.618 (0.411–0.930), *p* = 0.021] and almost longer than patients with IIIC/T4 disease [adjusted HR: 0.695 (0.471–1.025), *p* = 0.067]. Those with IIIC/T4 disease had similar survival than those with IVA disease [adjusted HR: 0.949 (0.669–1.345), *p* = 0.767], but those with IVA/T1–T3 disease have shorter survival than those with IIIC/T1–T3 disease [adjusted HR: 2.094 (1.792–2.446), *p* < 0.0005]. Also, those with IIIB/T3 disease had similar survival than those with IIIA disease [HR: 1.029 (0.828–1.280), *p* = 0.795].

From consecutive survival analyses, it was observed that for patients undergoing prostatectomy, only the TNM system, with the new N subdivision, and the Gleason grade group led to improved prognosis segregation. On the other hand, patients had refinement as a function of tumor size (T4) only. This enabled the formation of new staging criteria, as described in Table 1.

**Table 1:** New staging criteria proposed for prostate cancer patients.

When the patient…	And T is…	And N is…	And M is…	And PSA is…	And Gleason grade group is…	Then the stage is…
… received prostatectomy*	T2	Any	M0	Any	1–2	IA
	T2	Any	M0	Any	3–4	IB
	T3	N0	M0	Any	1–2	IB
	T3	N0	M0	Any	3	IIA
	T3	N1–N2	M0	Any	1–2	IIA
	T2	N0	M0	Any	5	IIB
	T3	N0–N2	M0	Any	4	IIB
	T3	N1–N3	M0	Any	3	IIB
	T4	N0	M0	Any	1–4	IIB
	T3	N0	M0	Any	5	IIC
	T2	N1–N3	M0	Any	5	IIIA
	T3	N1	M0	Any	5	IIIA
	T3	N3	M0	Any	4	IIIA
	T3	N2	M0	Any	5	IIIB
	T3	N3	M0	Any	5	IIIC
	T4	N1–N3	M0	Any	1–4	IIIC
	T4	Any	M0	Any	5	IIIC
… did not receive prostatectomy	cT1a-c, cT2a	N0	M0	< 10	1	IB
	pT2	N0	M0	< 10	1	IB
	cT1a-c, cT2a	N0	M0	< 10	1	IIA
	cT2b-c	N0	M0	≥ 10 < 20	1	IIA
	T1–T2	N0	M0	< 20	2	IIA
	T1–T2	N0	M0	< 20	3–4	IIB
	T1–T2	N0	M0	≥ 20	1–4	IIC
	T3	N0	M0	Any	1–4	IIC
	T1–T3	N0	M0	Any	5	IIIB
	T4	N0	M0	Any	1–4	IIIC
	T4	N0	M0	Any	5	IVA
	T4	N +	M0	Any	Any	IVA
Received or not prostatectomy	Any	Any	M1	Any	Any	IVB

*Refers to total or radical prostatectomy, not partial.

In general, the present reclassification depicts a moderate correlation with the current staging system (Spearman’s rho: 0.621, *p* < 0.0005; Kendall’s tau-b: 0.518, *p* < 0.0005]. However, this reclassification (Supplementary Table 5) [specific survival C-index: 0.908 (0.904–0.911); overall survival C-index: 0.769 (0.766–0.773)] outperformed the current one [specific survival C-index: 0.887 (0.883–0.891); overall survival C-index: 0.717 (0.714–0.721)] and eliminated the prognosis value of surgery observed in the model that contains the current staging system (not shown).

In order to validate such a classification system, patients from the SEER 8 registries were selected. A total of 147,719 patients were included in the first cohort, with a median follow-up of 84 months (0–191) and 29,219 deaths recorded, of which 39.1% (n = 11,486) were due to prostate cancer. In this cohort, again, this reclassification [specific survival C-index: 0.908 (0.906–0.911); overall survival C-index: 0.788 (0.786–0.791)] outperformed the current one [specific survival C-index: 0.892 (0.889–0.895); overall survival C-index: 0.744 (0.741–0.747)].

### Lymph nodes surgical approach and survival

Subsequently, we analyzed whether the number of resected lymph nodes has a prognosis value. The number of analyzed lymph nodes was used as a surrogate of resected lymph nodes.

It was observed that the larger the number of lymph nodes analyzed, the larger the survival in patients with N1 [HR: 0.97 (0.95–0.98), *p* < 0.001], N2 [HR: 0.95 (0.92–0.97), *p* < 0.001] and N3 [HR: 0.98 (0.96–0.99), *p* = 0.009] diseases, and in T3–T4/N0 disease as well [HR: 0.99 (0.98–1.00), *p* = 0.005].

**Figure 1:**
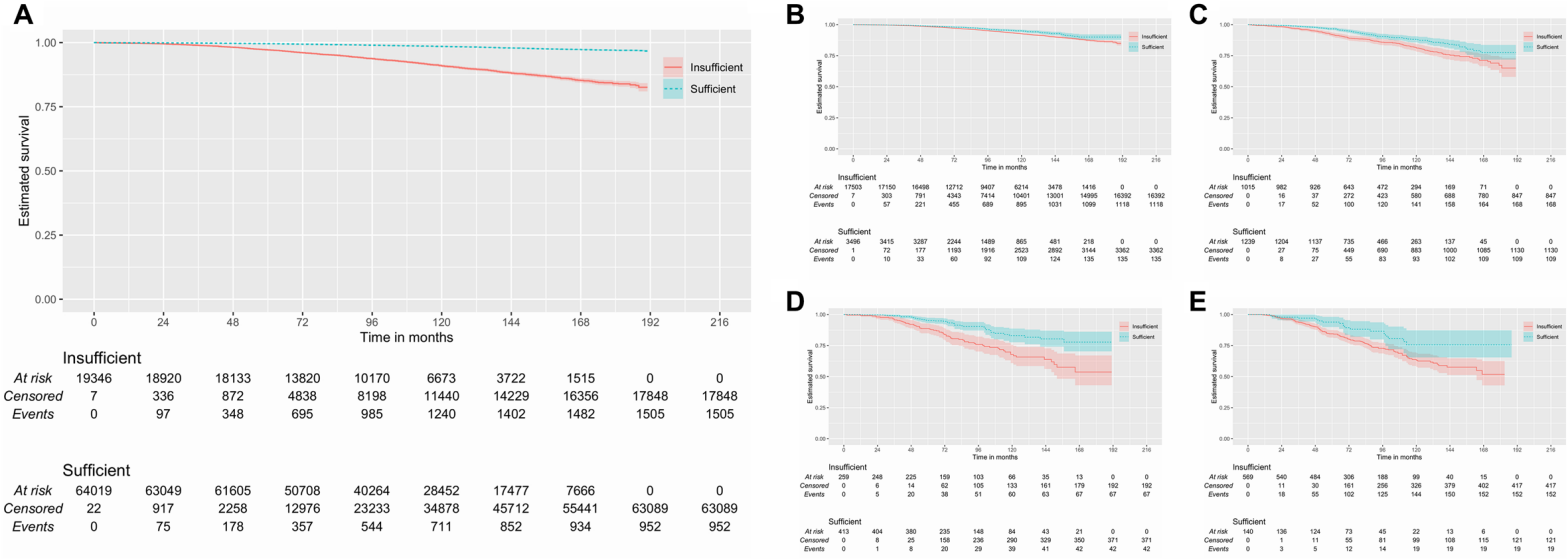
Kaplan–Meier plot of cancer specific survival according to the number of lymph nodes analyzed. (**A**) Analysis performed with all patients included (Log-Rank χ^2^: 2463.08; *p* < 0.0005; *n* = 83,394) and subgroup analysis of patients (**B**) T3-T4/N0 (Log-Rank χ^2^: 13.58; *p* < 0.0005; *n* = 21,007), (**C**) N1(1 +) (Log-Rank χ^2^: 17.17; *p* < 0.0005; *n* = 2254), (**D**) N2(2 +) (Log-Rank χ^2^: 20.91; *p* < 0.0005; *n* = 672), and (**E**) N3(> 2 +) (Log-Rank χ^2^: 8.58; *p* = 0.003; *n* = 709). The 95% confidence intervals are represented by the colored outline around the curves.

After reclassifying the dissection based on cutoffs obtained in the analysis (T3–T4/N0: > 12; N1: > 7; N2: > 8; N3: > 23), the 15-year survival analysis shows a progressively greater difference in these subgroups (Fig. 1). Specifically, this difference is almost 3% in patients with T3–T4/N0 disease [insufficient: 86.4% (85.3–87.4%); sufficient 90.0% (87.8–92.2%)], almost 10% in patients with N1 disease [insufficient: 67.3% (61.1–74.1%); sufficient: 77.6% (71.9–83.7%)], and greater than 20% in patients with N2 [insufficient: 53.7% (43.1–67.1%); sufficient: 77.8% (70.3–86.1%)] and N3 diseases [insufficient: 51.8% (43.0–62.5%); sufficient: 75.6% (65.5–87.3%)].

The sufficient resection resulted in being an independent prognosis factor [HR: 0.183 (0.157–0.213), *p* < 0.0005], with a 39% reduction in death risk, compared to those who received insufficient resection in the period analyzed, after adjustment for age, race, histology, Gleason grade group, PSA, T and number of metastatic lymph nodes [adjusted HR: 0.610 (0.498–0.747), *p* < 0.0005].

Although an increasing median of lymph nodes analyzed was observed in T3–T4/N0 [adjusted IRR: 1.100 (1.086–1.113), *p* < 0.0005], N1 [adjusted IRR: 1.536 (1.488–1.586), *p* < 0.0005], N2 [adjusted IRR: 1.861 (1.759–1.970), *p* < 0.0005] and N3 [adjusted IRR: 2.430 (2.302–2.566), *p* < 0.0005] groups compared to T2/N0 group, only patients with N1 [OR: 6.162 (5.620–6.757), *p* < 0.0005] and N2 [OR: 7.997 (6.800–9.404), *p* < 0.0005] had greater odds of receiving a procedure with dissection of sufficient lymph nodes, while those with larger lymph node burden (> 2 +) did not [OR: 1.159 (0.959–1.402), *p* = 0.127].

Association analysis, again excluding T2/N0 patients, shows a strong negative association between T3–T4/N0 disease and a sufficient resection, but a strong positive between this type of procedure and N1 and N2 diseases, with a moderate effect (Cramer’s V: 0.314, *p* < 0.0005). No association was observed with N3 diseases.

Despite an important tendency to increase the dissection towards enough lymph nodes after 2009 [adjusted OR: 1.883 (1.757–2.019), *p* < 0.0005], the group of patients with N3 disease continued to be characterized by an insufficient resection in the period 2010–2015 (not shown).

### Lymph node resection and staging

**Figure 2:**
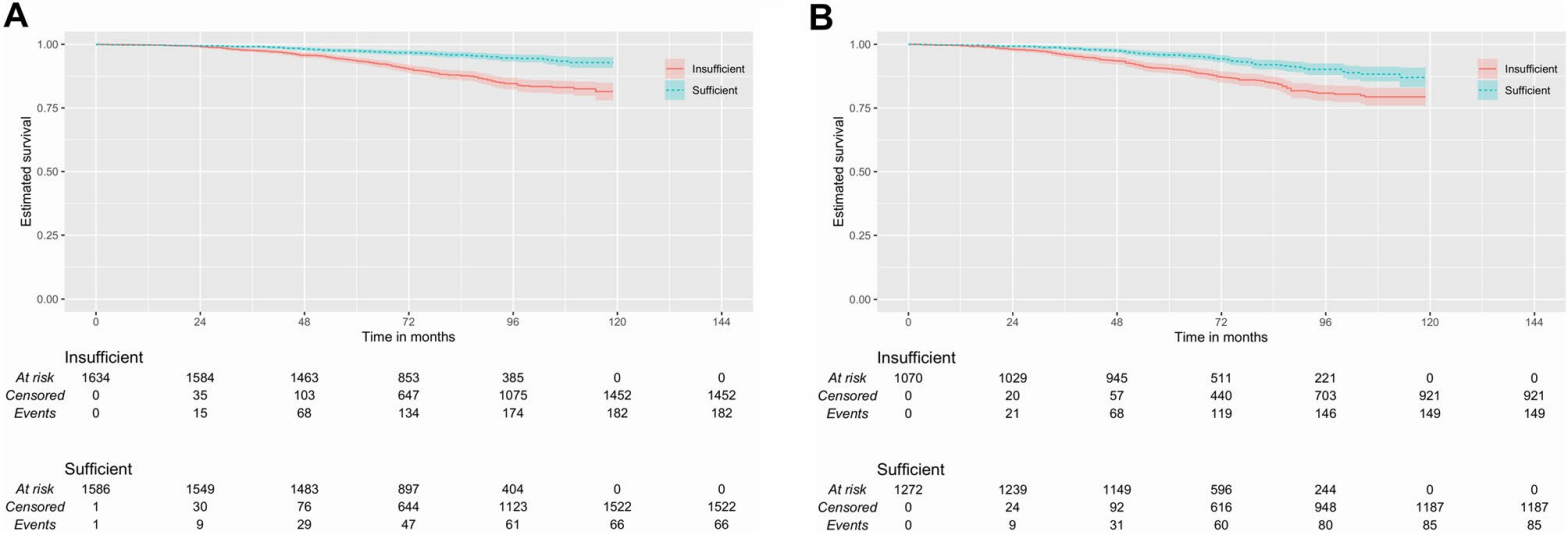
Kaplan–Meier plot of cancer specific survival according to the number of lymph nodes analyzed in patients staged by the AJCC 8th edition manual. Analysis performed in patients of the stage IIIC (Log-Rank χ^2^: 56.60; *p* < 0.0005; *n* = 3222) (**A**) and IVA (**B**) (Log-Rank χ^2^: 29.97; *p* < 0.0005; *n* = 2342). The 95% confidence intervals are represented by the colored outline around the curves.

Finally, we tested whether the resection has the potential to modify the staging criteria. Starting with the staging of the 8th edition of the AJCC, one could observe a clinically relevant difference in stages IIIC and IVA (Fig. 2).

As this association between the number of resected lymph nodes and survival may be an indirect effect of the number of affected lymph nodes, which is not contemplated in the current staging system, multivariable models including this factor were developed. Only patients with stage IVA were included in these analyses, as those with stage IIIC disease do not have lymph node metastasis (N0).

In patients with IVA disease, the multivariable model without inclusion of lymph node metastasis categories showed the number of resected lymph nodes as an independent diagnostic factor after correcting for histology, race, T, Gleason grade group and PSA levels [sufficient vs. insufficient, adjusted HR: 0.533 (0.404–0.703), *p* < 0.0005], remaining after inclusion of lymph node metastasis categories [sufficient vs. insufficient, adjusted HR: 0.591 (0.440–0.794), *p* < 0.0005].

As for the restaging obtained in the previous analyses, even observing some difference in survival for stages IIA (Log-Rank χ^2^: 3.93; *p* = 0.048), IIIB (Log-Rank χ^2^: 4.92; *p* = 0.027) and IIIC (Log-Rank χ^2^: 4.17; *p* = 0.041), such differences are not clinically relevant due to either the difference in total mortality observed, or the number of patients allocated in each rating (insufficient vs. sufficient) (not shown). However, for the new stage IIC classification, a significant difference is observed with a reasonable number of patients, with a mortality rate of more than 5% lower in those who received a sufficient resection (Supplementary Fig. 5); such patients have survival that is not different from stage IIB patients [IIB vs. IIC/sufficient, adjusted HR: 0.680 (0.431–1.073), *p* = 0.097].

## Discussion

The superiority of the current staging method has been corroborated^10–13^, with few studies proposing changes^14^, and no study up to the present date has analyzed the prognosis difference as a function of prostatectomy and the number of metastatic lymph nodes. Although some investigations have shown an important prognosis value in the number of lymph nodes affected^15–18^, a greater value classification was reached in this study by not simply dichotomizing the N factor in N− and N + even if it involves a different cutoff point of affected lymph nodes, being relevant for restaging.

Interestingly, a previous study using SEER and the National Cancer Data Base (NCDB) obtained results close to ours regarding N stratification with differential prognosis depending on the number of affected lymph nodes. Despite having observed an additional category for the stratification of N, the first three of this study coincide with the first three of the present study, reinforcing the current findings. Probably due to the greater number of patients with complete data, this study was able to find one more category with independent prognostic value. However, even proposing, they did not carry out a restaging and were limited to analyzing the patients submitted to prostatectomy^18^. In this study, one could observe that lymph node metastasis has a dual prognosis value depending on the performance of the surgery, observing that the metastatic burden has a very high prognosis weight in patients submitted to prostatectomy, implying an improvement in the identification of patients that are more likely to die. This was confirmed by the difference in Harrell's C-index of this new classification with the current one of the AJCC. To our understanding, this is the first study to report that.

Some studies have shown the importance of lymph node resection in patients with prostate cancer, both with lymph node metastasis^17,19–21^ and those at intermediate-to-high risk without lymph node metastasis^22^, which is in line with current guidelines^23,24^. In agreement, we observed that the greater number of lymph nodes analyzed is associated with greater survival.

As observed in this study, the ideal number of resected lymph nodes depends on the number affected and has a direct impact on survival. Based on this, it could be concluded that an intraoperative analysis of the lymph nodes would be important for expanding the approach. However, current prostate cancer treatment guidelines do not recommend such a procedure due to the complications that the increase in procedure time generates^23–26^. Therefore, most recommend analyzing the risk of the patient having a lymph node metastasis and the risk of the disease. If a pelvic lymph node dissection (PLND) is found to be necessary, the extended approach is currently recommended^23–26^.

Pre-surgical identification of the extent of PLND currently involves the use of nomograms that combine clinicopathological factors, including imaging tests^3,23–26^. Even though we observed a significant increase in the prevalence of procedures with enough dissected lymph nodes over time, it can be observed that the identification of patients who derive greater benefit from an extended or super-extended PLND (N3) does not receive it. Notwithstanding, a previous study showed that patients with high-risk disease who may benefit from a super-extended PLND can be identified with the nomograms themselves, but with a higher cutoff point in the risk for metastasis when compared to those currently advocated by some guidelines (30% vs. 5% or 7%)^23,24,27^. Similarly, another study observed that risk greater than 60% better identified patients with benefit from extended PLND, most of whom had non-regional metastasis^28^. Therefore, the present results reinforce the need for a more refined recommendation of the extension of the PLND in function of the risk of lymph node metastasis and, perhaps, intraoperative analysis of the lymph nodes in selected patients in order to perform a super-extended approach when necessary.

Furthermore, most patients who would benefit from, at least, an extended or standard PLND (T3–T4/N0, N1 and N2 diseases) do not receive it either, with an excess of mortality in these patients in the long term. Therefore, there is no justification for limiting the extension of the PLND, as corroborated by the literature data, including a randomized clinical trial^29,30^. In this regard, one of the main findings is the association between survival and the number of resected lymph nodes in patients with T3–T4 disease, even in the absence of metastatic lymph nodes. Most likely, this phenomenon can be explained by downstaging due to the failure to remove the affected lymph nodes, as described in the literature^31,32^. Even in patients with affected lymph nodes, a limited or standard PLND may imply downstaging, since more than half of these patients have metastasis in lymph nodes of the internal iliac chain^33^, which is removed in an extended or super-extended PLND^26^. Such results reinforce the need for a careful analysis of the risk of lymph node metastasis to approach the correct number of lymph node chains in order not to impair the survival of patients^34^, even in the absence of metastatic lymph nodes.

An interesting finding was the difference in the degree of association between the adequate number of lymph nodes and specific survival in the current staging categories and the new proposal, obviously considering only patients undergoing prostatectomy. This contrasts with the analyses made by subgroups divided by pT and pN. A possible explanation is precisely the other staging classification criteria, specifically the Gleason grade group. Probably, the restaging proposed groups patients with closer characteristics, increasing the homogeneity of the characteristics. Since the Gleason grade group has a direct relationship with survival, and the same is used for risk classification and therapeutic indications, it is possible that patients, even with inadequate resection of lymph nodes, but with other less aggressive characteristics of the disease, do not derive as much benefit as those with a more aggressive disease. However, no analysis was performed to test whether there is a change in the effect of the number of resected lymph nodes on survival due to other characteristics. Therefore, we are unable to prove that.

This study has some important limitations. The main one concerns the classification of the extension of PLND. It is important to highlight that the extent of PLND is classified by the removed lymph node chains and not by their dissected amount^26^. Even though, logically, the increase in removed chains implies a greater number of removed lymph nodes and, consequently, the number of lymph nodes analyzed^35^, this limitation of the study must be considered since the latter was available in the database.

The retrospective nature also implies limitations and possible selection bias, requiring validation with other cohorts, mainly prospective ones. However, it was possible to carry out a validation with another SEER Program database with less stringent exclusion criteria, which corroborated the initial findings.

In addition, therapeutic recommendations have changed over time and, with increasing life expectancy and improvements in diagnosis, surgery can be de-escalated, active surveillance being recommended instead^36^. However, staging according to the criteria found in this study provided a higher concordance index (C-index), even not taking this criterion into account.

Finally, we mention the adjuvant treatments performed. We did not have access to adjuvant treatments, such as radiotherapy, which may imply an important prognostic change, even though the reporting of such treatments in SEER has a non-high accuracy^37,38^. However, the multivariable analyses partialized the influence of the main confounders. Thus, the association of lymph node resection with survival should be understood as carrying the average effect of the other treatments performed that were not included in the models, being necessary corroboration by studies that also partialize the effects of these adjuvant therapies. Previous studies have shown that the addition of adjuvant radiotherapy to standard androgen deprivation treatment in patients with pN1 disease depends primarily on the number of metastatic lymph nodes. Improved overall survival was observed with the addition of radiotherapy in patients with 3 or 4 positive lymph nodes, or with up to 2 positive lymph nodes when they had pT3(b)-T4 disease with a Gleason score 7–10, but no extra benefit in other patients, especially in those with more than 4 affected lymph nodes^4,5^. Because these characteristics are very similar to the proposed reclassification, this staging reclassification may even help in directing therapeutic recommendations.

## Conclusion

Prostate cancer staging should follow different criteria for patients undergoing prostatectomy from those with only clinical evaluation, since the N factor has a modified prognostic value depending on the surgery and could be stratified according to the number of affected lymph nodes, as validated in an independent cohort.

## Supplementary Information

Supplementary Information.

## Data Availability

The data that support the findings of this study are available from the National Cancer Institute Surveillance, Epidemiology and End Results Program but restrictions apply to the availability of these data, which were used under license for the current study, and so are not publicly available. Data are however available from the authors upon reasonable request and with permission of the National Cancer Institute Surveillance, Epidemiology and End Results Program. For any inquiry regarding data and materials, please contact the corresponding author FAC Luz.
