# Supplementary material for: Analysis of the surgical approach in prostate cancer staging: results from the surveillance, epidemiology and end results program
Source: Sci Rep. 2023 Jun 19;13:9949. doi: 10.1038/s41598-023-37204-y (PMC10279689; doi:10.1038/s41598-023-37204-y)
Supplement: Supplementary file 1 — Supplementary Information. [file 41598_2023_37204_MOESM1_ESM.docx]

**Supplementary Material for “Analysis of the surgical approach in prostate cancer staging: results from the Surveillance, Epidemiology and End Results Program”**

Felipe Andrés Cordero da Luz^1,2*^, Camila Piqui Nascimento^1^, Eduarda da Costa Marinho^1^, Pollyana Júnia Felicidade^1^, Rafael Mathias Antonioli^1^, Rogério Agenor de Araújo^1,2,3^, and Marcelo José Barbosa Silva^3^.

^1^Center for Cancer Prevention and Research, Uberlandia Cancer Hospital, Av Amazonas nº 1996, Umuarama, Uberlândia, Minas Gerais, MG 38405-302, Brazil

^2^Laboratory of Tumor Biomarkers and Osteoimmunology, Department of Immunology, Institute of Biomedical Sciences, Federal University of Uberlandia, Av Pará nº 1720, Bloco 6T, room 07, Umuarama, Uberlândia, Minas Gerais, 38.405-320, Brazil

^3^Medical Faculty, Federal University of Uberlandia, Av Pará nº 1720, Bloco 2U, Umuarama, Uberlândia, Minas Gerais, MG 38400-902, Brazil

^*^Correspondence:

Corresponding author: Felipe Andrés Cordero da Luz, Ph.D., Center for Cancer Prevention and Research, Uberlandia Cancer Hospital, Minas Gerais, Brazil.

Av. Amazonas 1996 CEP: 38405-302

Phone: +55 (34)3291-6166;

Email: [felipe.cordero@ufu.br](mailto:felipe.cordero@ufu.br)**;** felipecorderodaluz@gmail.com.

For any inquiry regarding data and materials, please contact the corresponding author FAC Luz.

**Supplementary Tables**

**Supplementary Table 1. Prognostic value by Cox regression of lymph node metastasis in prostate cancer patients (M0) submitted to prostatectomy (*n* = 39,925).**

| **Cancer specific survival** | | | | |
| --- | --- | --- | --- | --- |
|  | **Univariable** | | **Multivariable^*^** | |
| **Comparison** | **HR (95%CI)** | ***p*** | **HR (95%CI)** | ***p*** |
| **Lymph node metastasis** |  | <0.0005 |  | <0.0005 |
| N0 | 1 |  | 1 |  |
| N1(1+) | 6.406 (5.169– 7.939) | <0.0005 | 1.900 (1.522 – 2.372) | <0.0005 |
| N2(2+) | 9.995 (7.362 – 13.570) | <0.0005 | 2.199 (1.607 – 3.009) | <0.0005 |
| N3(3-5+)^#^ | 13.818 (10.629 – 18.595) | <0.0005 | 2.817 (2.069 – 3.837) | <0.0005 |
| N4(>5+)^#^ | 24.572 (16.779 – 35.985) | <0.0005 | 4.304 (2.900 – 6.389) | <0.0005 |

^*^Stepwise Forward LR method; last step model adjusted by age (<70 years, 70-79 years, 80-84 years and 85+ years), race, PSA (<10 ng/mL vs. ≥10 ng/mL), T (T2, T3, and T4), histology groups and Gleason grade group.

^#^No statistical difference

**Supplementary Table 2. Univariable Cox regression of lymph node metastasis in prostate cancer patients submitted to prostatectomy according to T stage.**

| **Cancer specific survival** | | | | | | |
| --- | --- | --- | --- | --- | --- | --- |
|  | **T2 (*n* = 59,412)** | | **T3 (*n* = 23,204)** | | **T4 (*n* = 778)** | |
| **Comparison** | **HR (95%CI)** | ***p*** | **HR (95%CI)** | ***p*** | **HR (95%CI)** | ***p*** |
| **Lymph node metastasis** |  | <0.0005 |  | <0.0005 |  | <0.0005 |
| N0 vs. N1(1+) | 0.117 (0.084 – 0.164) | <0.0005 | 0.356 (0.309 – 0.411) | <0.0005 | 0.456 (0.273 – 0.759) | 0.003 |
| N1(1+) vs. N2(2+) | 0.865 (0.385 – 1.943) | 0.725 | 0.795 (0.619 – 1.020) | 0.071 | 0.568 (0.293 – 1.103) | 0.095 |
| N2(2+) vs. N3(>2+) | 0.441 (0.180 – 1.081) | 0.074 | 0.589 (0.449 – 0.774) | <0.0005 | 0.851 (0.454 – 1.596) | 0.615 |

**Supplementary Table 3. Three-, 5- and 10-year survival of non-metastatic (M0) prostate cancer patients submitted to prostatectomy according to T and N (*n* = 83,394).**

| **Year** | **T and N** | **Number at risk** | **Number of events** | **Survival (95% CI)** |
| --- | --- | --- | --- | --- |
| 3 | T2/N0 | 57,550 | 73 | 99.9% (99.8% - 99.9%) |
|  | T2/N1 | 484 | 3 | 99.4% (98.7% - 100.0%) |
|  | T2/N2 | 84 | 1 | 98.8% (96.6% - 100.0%) |
|  | T2/N3 | 77 | 2 | 97.5% (94.1% - 100.0%) |
|  | T3/N0 | 19,773 | 126 | 99.4% (99.3% - 99.5%) |
|  | T3/N1 | 1,601 | 40 | 97.6% (96.9% - 98.3%) |
|  | T3/N2 | 521 | 10 | 98.1% (97.0% - 99.3%) |
|  | T3/N3 | 525 | 30 | 94.6% (92.7% - 96.5%) |
|  | T4/N0 | 568 | 14 | 97.6% (96.4% - 98.9%) |
|  | T4/N1 | 65 | 7 | 90.2% (83.5% - 97.4%) |
|  | T4/N2 | 36 | 4 | 90.1% (81.3% - 99.8%) |
|  | T4/N3 | 53 | 7 | 88.6% (81.1% - 96.9%) |
|  |  |  |  |  |
| 5 | T2/N0 | 52,211 | 82 | 99.7% (99.7% - 99.8%) |
|  | T2/N1 | 405 | 8 | 97.7% (96.3% - 99.0%) |
|  | T2/N2 | 72 | 1 | 97.7% (94.5% - 100.0%) |
|  | T2/N3 | 68 | 3 | 93.6% (88.3% - 99.2%) |
|  | T3/N0 | 16,839 | 215 | 98.2% (98.1% - 98.4%) |
|  | T3/N1 | 1,251 | 56 | 93.9% (92.7% - 95.1%) |
|  | T3/N2 | 392 | 27 | 92.7% (90.5% - 95.0%) |
|  | T3/N3 | 368 | 43 | 86.3% (83.4% - 89.3%) |
|  | T4/N0 | 524 | 20 | 94.1% (92.2% - 96.1%) |
|  | T4/N1 | 57 | 3 | 86.0% (78.2% - 94.4%) |
|  | T4/N2 | 32 | 3 | 82.4% (71.4% - 95.1%) |
|  | T4/N3 | 38 | 7 | 76.9% (66.9% - 88.3%) |
|  |  |  |  |  |
| 10 | T2/N0 | 27,657 | 296 | 99.0% (98.9% - 99.1%) |
|  | T2/N1 | 156 | 18 | 91.3% (88.2% - 94.6%) |
|  | T2/N2 | 26 | 5 | 85.5% (75.3% - 97.1%) |
|  | T2/N3 | 23 | 9 | 75.2% (64.2% - 88.1%) |
|  | T3/N0 | 6,854 | 596 | 93.1% (92.7% - 93.6%) |
|  | T3/N1 | 384 | 95 | 82.8% (80.4% - 85.3%) |
|  | T3/N2 | 109 | 41 | 76.3% (71.3% - 81.7%) |
|  | T3/N3 | 91 | 53 | 65.7% (60.3% - 71.7%) |
|  | T4/N0 | 369 | 33 | 87.3% (84.5% - 90.2%) |
|  | T4/N1 | 32 | 4 | 78.5% (68.9% - 89.4%) |
|  | T4/N2 | 17 | 7 | 60.6% (46.1% - 79.7%) |
|  | T4/N3 | 11 | 9 | 50.5% (36.5% - 69.9%) |

**Supplementary Table 4. Prognosis value of staging by the proposed new criteria (*n* = 170,812).**

| **Cancer specific survival** | | | | |
| --- | --- | --- | --- | --- |
|  | **Univariable** | | **Multivariable^*^** | |
| **Variable** | **HR (95%CI)** | ***p*** | **HR (95%CI)** | ***p*** |
| **Stage by new classification criteria** |  | <0.0005 |  | <0.0005 |
| IA | 1 |  | 1 |  |
| IB | 3.314 (2.636 – 4.165) | <0.0005 | 3.184 (2.533 – 4.003) | <0.0005 |
| IIA | 8.593 (6.878 – 10.736) | <0.0005 | 7.764 (6.211 – 9.705) | <0.0005 |
| IIB | 19.631 (15.795 – 24.398) | <0.0005 | 17.256 (13.873 – 21.462) | <0.0005 |
| IIC | 42.957 (34.546 – 53.415) | <0.0005 | 36.479 (29.313 – 45.399) | <0.0005 |
| IIIA | 71.588 (53.403 – 95.965) | <0.0005 | 70.618 (52.676 – 94.670) | <0.0005 |
| IIIB | 111.926 (90.404 – 138.572) | <0.0005 | 88.747 (71.576 – 110.037) | <0.0005 |
| IIIC | 156.778 (124.842 – 196.885) | <0.0005 | 143.049 (113.866 – 179.712) | <0.0005 |
| IVA | 289.696 (221.090 – 379.590) | <0.0005 | 241.278 (183.994 – 316.397) | <0.0005 |
| IVB | 631.877 (512.452 – 779.132) | <0.0005 | 530.844 (430.137 – 655.128) | <0.0005 |

^*^Adjusted by age (<70 years, 70-79 years, 80-84 years and ≥85 years), race, median household income and histology.

**Supplementary Figures**


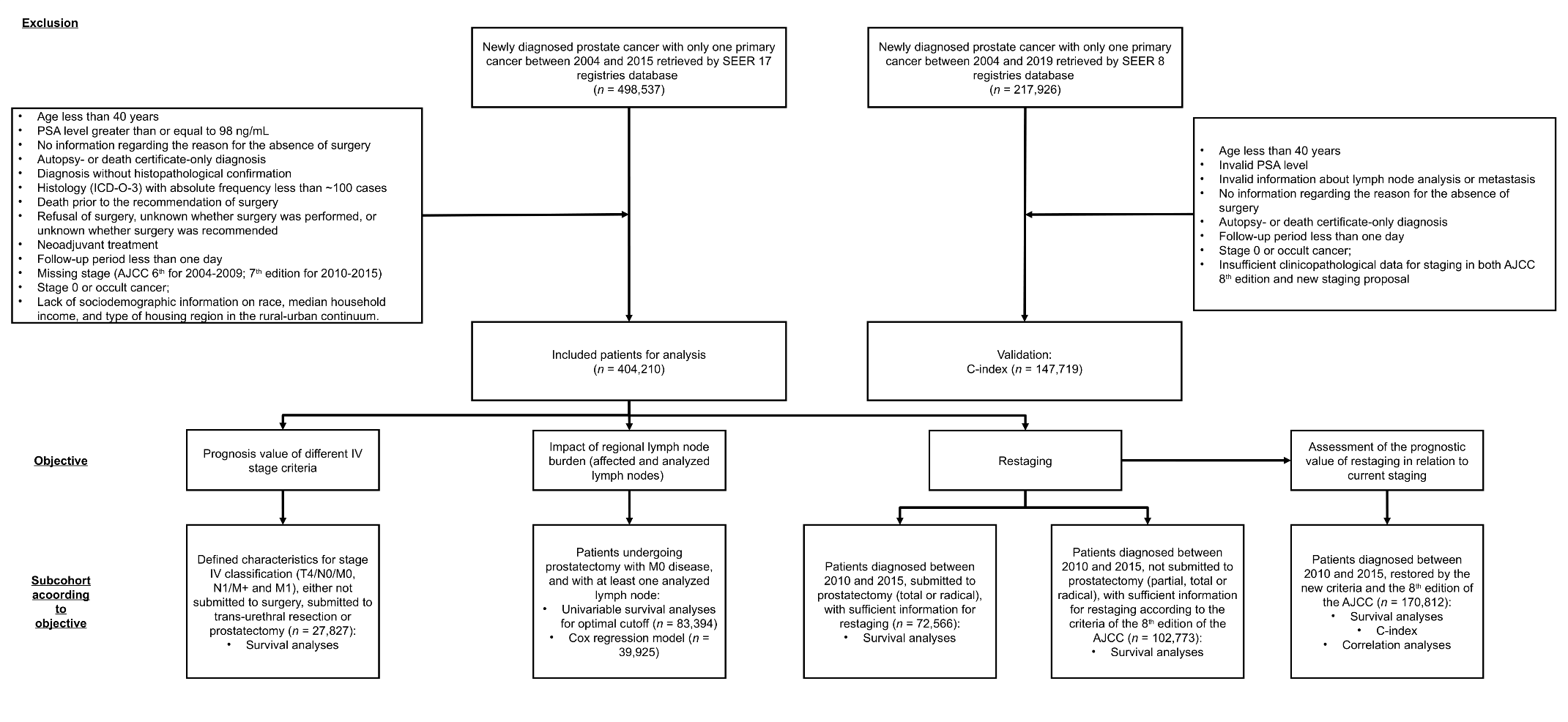


**Supplementary Figure 1. Flowchart of patients included and excluded in each statistical analysis.**


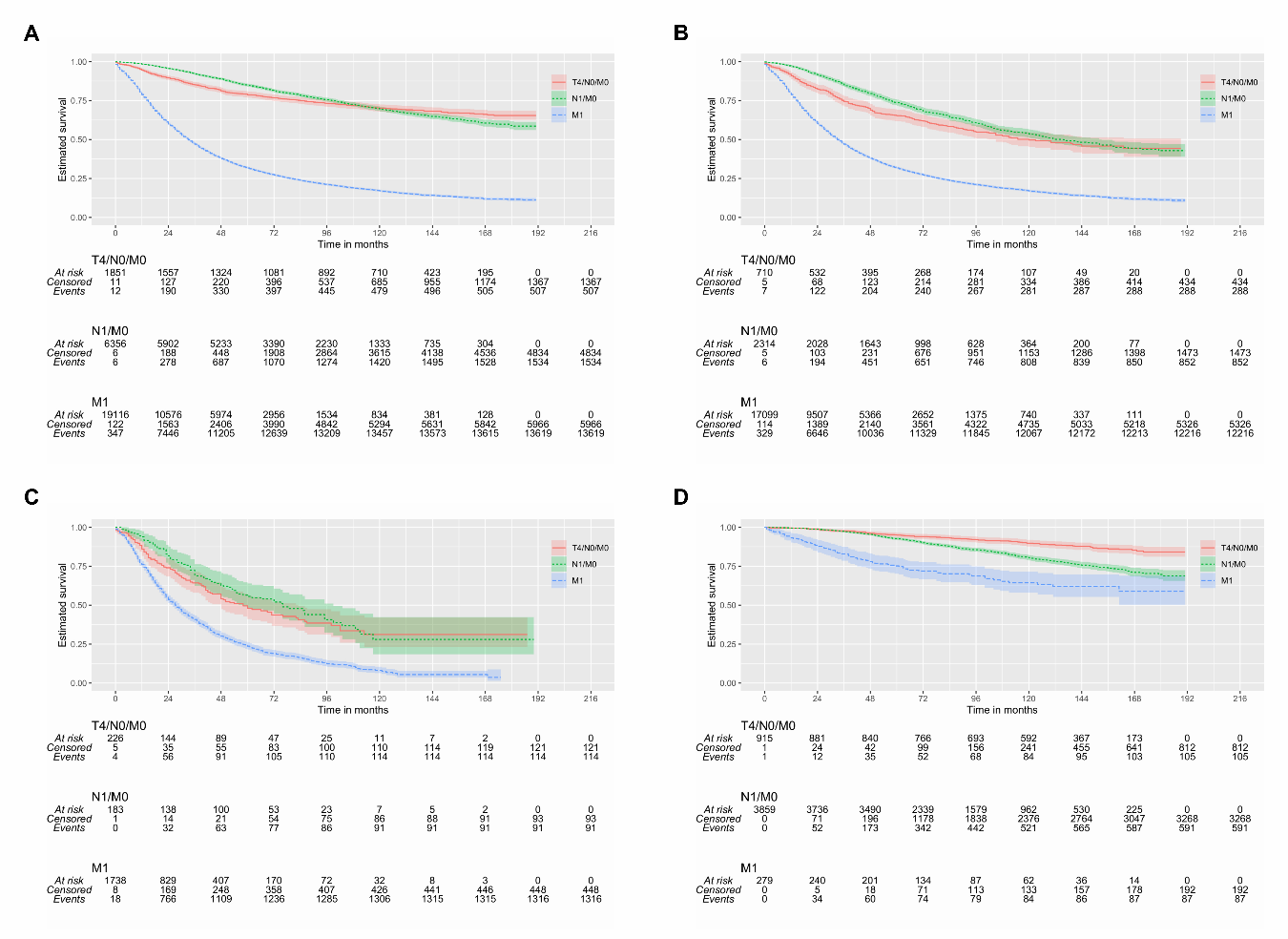


**Supplementary Figure 2. Kaplan-Meier plot of cancer specific survival by stage IV criteria of the AJCC 7^th^ edition.** All included stage IV patients (A) (Log-Rank χ^2^: 5,556.97, p<0.0005; *n*=22,232). Patients who did not receive a surgical procedure (B) (Log-Rank χ^2^: 1,386.91, p<0.0005; *n*=15,342). Patients who received trans-urethral resection (C) (Log-Rank χ^2^: 110.86, p<0.0005; *n*=1,741). Patients who received prostatectomy (D) (Log-Rank χ^2^: 139.22, p<0.0005; *n*=4,908). The 95% confidence intervals are represented by the colored outline around the curves.


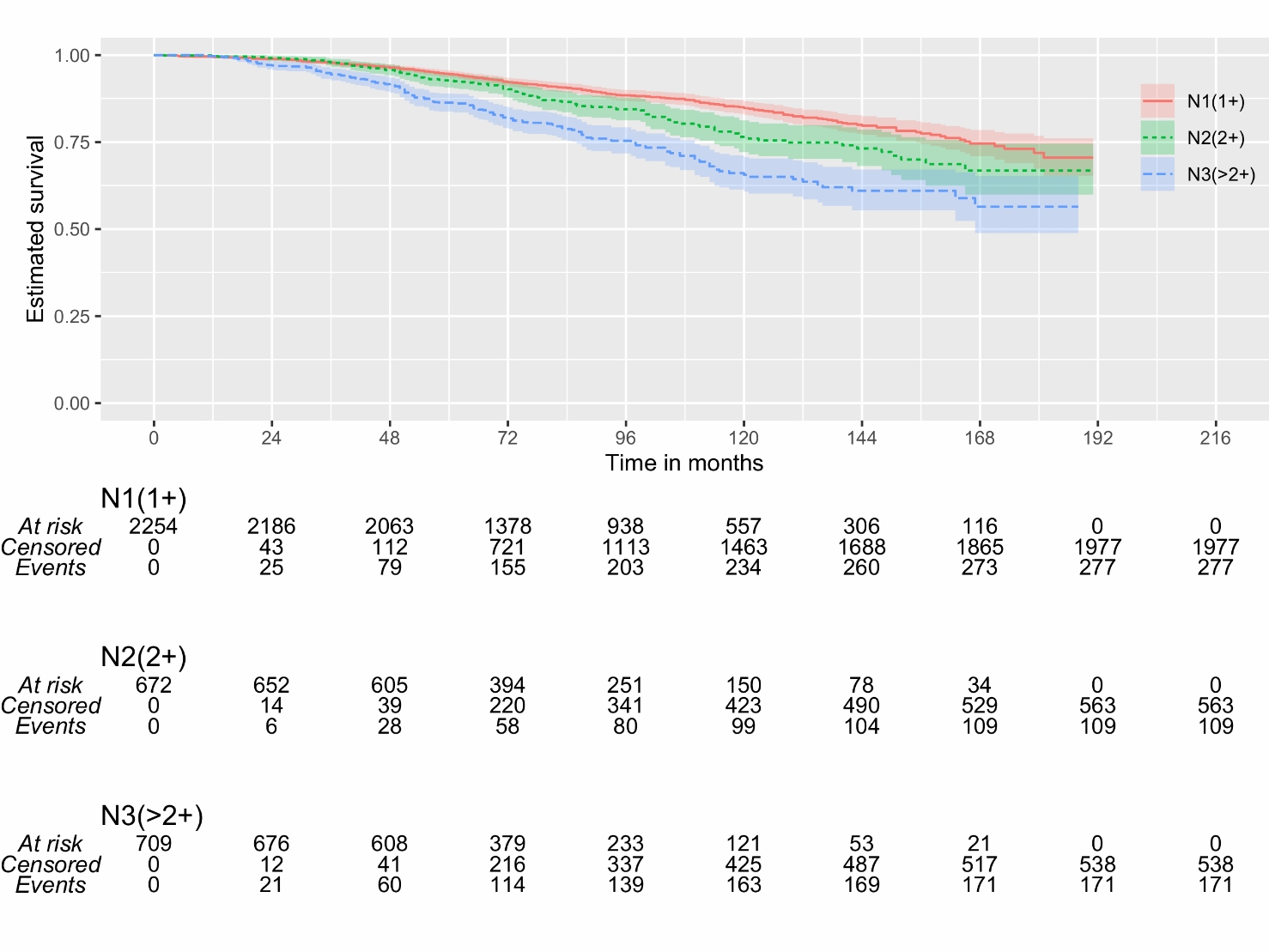


**Supplementary Figure 3.** **Kaplan-Meier plot of cancer specific survival by reclassification according to lymph node metastasis in patients submitted to prostatectomy and N+ disease.** Log-Rank χ^2^: 78.28; p<0.0005 (*n*=3,635). The 95% confidence intervals are represented by the colored outline around the curves.


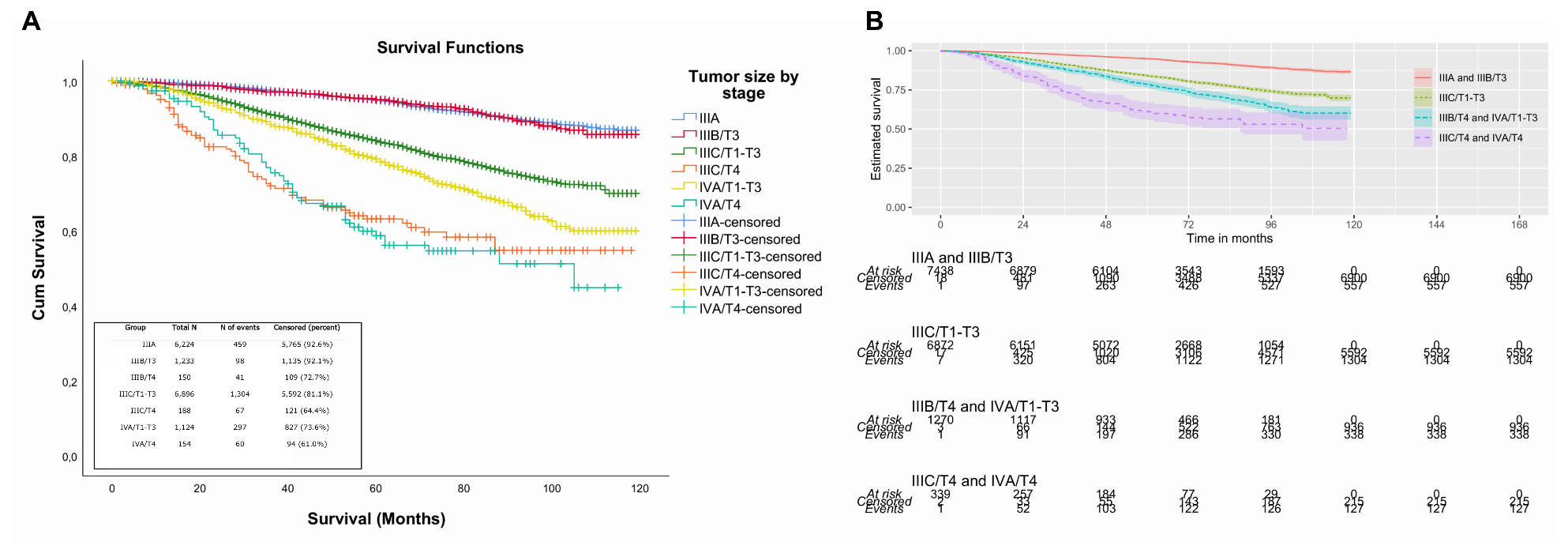


**Supplementary Figure 4. Kaplan-Meier plot of cancer specific survival in clinically staged prostate cancer patients with IIIA to IVA disease according to tumor size (T).** (A) The analysis of the prognostic value of tumor size showed an important segregation according to the T4 category (Log-Rank χ^2^: 824.23, p<0.0005), (B) allowing to gather subdivisions into more homogeneous groups (Log-Rank χ^2^: 821.53, p<0.0005). A total of 15,696 patients were included in these analyses. The 95% confidence intervals are represented by the colored outline around the curves (Panel B).


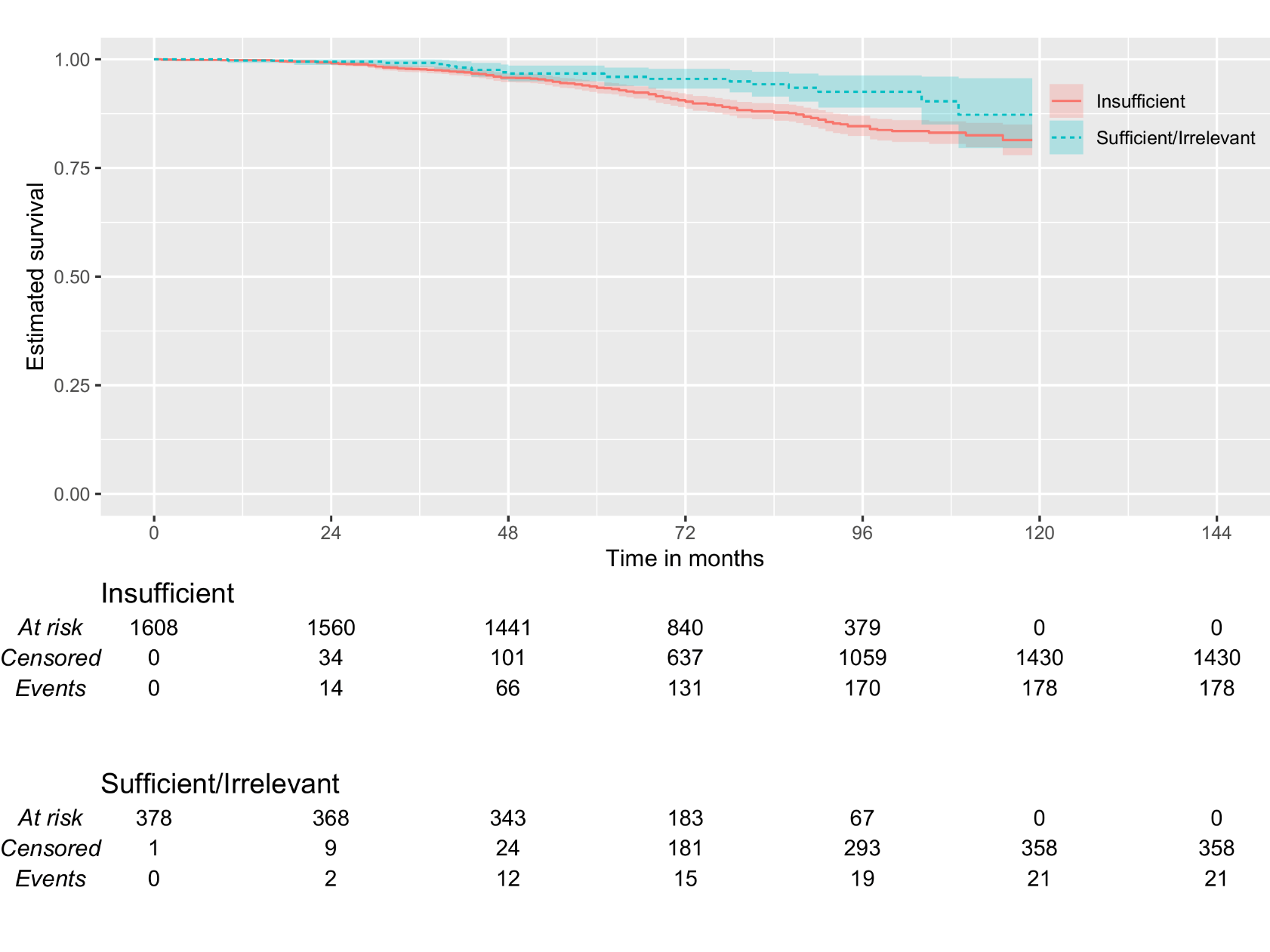


**Supplementary Figure 5.** **Kaplan-Meier plot of cancer specific survival according to the number of lymph nodes analyzed in patients staged as IIC in the proposed staging criteria.** Log-Rank χ^2^: 7.81, p=0.005; *n*=1,987. The 95% confidence intervals are represented by the colored outline around the curves.
